# Correlation between Prostatitis, Benign Prostatic Hyperplasia and Prostate Cancer: A systematic review and Meta-analysis

**DOI:** 10.7150/jca.37235

**Published:** 2020-01-01

**Authors:** Lei Zhang, Yi Wang, Zhiqiang Qin, Xian Gao, Qianwei Xing, Ran Li, Wei Wang, Ninghong Song, Wei Zhang

**Affiliations:** 1 Department of Urology, The First Affiliated Hospital of Nanjing Medical University, Nanjing, 210009, China.; 2 Department of Urology, Nanjing First Hospital, Nanjing Medical University, Nanjing, 210006, China.; 3 Department of Oncology, The First Affiliated Hospital of Nanjing Medical University, Nanjing, 210009, China.; 4 Department of Urology, The Affiliated Hospital of Nantong University, Nantong, 226001, China.

**Keywords:** Prostatitis, Benign prostatic hyperplasia, Prostate cancer, Meta-analysis, Epidemiological.

## Abstract

**Background**: No consensus has been reached on the definite associations among prostatitis, benign prostatic hyperplasia (BPH) and prostate cancer (PCa). Hence, this meta-analysis was conducted to explore their triadic relation by summarizing epidemiological evidence.

**Methods**: Systematical and comprehensive retrieval of online databases PubMed, PMC, EMBASE and Web of Science was performed to acquire eligible studies, up to April 1st, 2019. Pooled odds ratios (ORs) with 95% confidence intervals (CIs) were calculated to clarify their correlations.

**Results**: A total of 42 studies were enrolled in the quality assessment and 35 were finally included in the meta-analyses. Among them, 27 studies were included to describe the association between prostatitis and PCa (OR=1.72, 95% CI=1.44-2.06, *I^2^*=90.1%, *P*<0.001). 21 studies presented significant evidence about the relation between BPH and PCa (OR=2.16, 95% CI=1.75-2.88, *I²*=97.1%, *P*<0.001). Due to the huge heterogeneity among studies, those with obvious outliers were excluded based on the Galbraith plots. Ultimately, 17 studies were screened out to assess the association between prostatitis and PCa (OR=1.59, 95% CI=1.48-1.70, *I²*=29.4%, *P*=0.123). Meanwhile, 8 studies were retained to evaluate the association between BPH and PCa (OR=3.10, 95% CI=2.87-3.35, *I²*=8.4%, *P*=0.365). As for the relation between prostatitis and BPH, a case-control study and a cohort study both supported that prostatitis could enhance the risk of BPH.

**Conclusions**: Significant correlations were revealed among prostatitis, BPH and PCa. Prostatitis or BPH could lead to escalating risks of PCa. Meanwhile, people with a history of prostatitis might be more vulnerable to BPH.

## Introduction

Prostate cancer (PCa) is the most prevalent malignancy in the male population, with 164,690 newly estimated cases and 29,430 newly estimated deaths in the United States, 2018 1. PCa mainly occurs in elderly men, and nearly two thirds of cases are diagnosed at the age of 65 or over 2. Up to now, several factors have been verified associated with the carcinogenesis of PCa, including aging, family history and race 3-5. Furthermore, the altered androgen metabolism also plays a pivotal role 6. However, the definite etiological mechanism remains unclear. With the assistance of epidemiological studies, a large number of potential risk factors for PCa, such as work environment, obesity, smoking, alcohol consumption and sexual activity, have been identified in the past decades 7-11. Unexpectedly, prostatitis and benign prostatic hyperplasia (BPH) were listed among these risk factors, though controversies existed 12, 13.

Prostatitis and BPH are two common benign diseases of the prostate gland. Prostatitis affects men of all ages, especially the middle age group. Both of them have high incidence ranging from 3 to 16% in Europe, North America and Asia 14-16. Moreover, more than 50% of the surgical prostate specimens were found to be associated with histological inflammation 17. Pathogenesis of prostatitis includes pathogens infection like bacteria and mycoplasma, urine reflux, autoimmunity, neuro muscular mechanisms and so on 18-20. However, BPH predominantly occurs in elderly men, and 70% of the patients are 70 years old or over 21. Aging and androgen are established factors leading to the occurrence of BPH 22. Furthermore, metabolic syndrome, genetics and lifestyle may also have something to do with BPH 23, 24. As for the correlation between prostatitis and BPH, Adorini et al. suggested a significant role of inflammation in the occurrence and progression in BPH 25. What's more, Jennifer et al. found an increased risk of BPH in those men with a history of prostatitis 26. However, it has yet to be further determined.

As a clinician, we are often enquired of by the anxious patients with prostatitis or BPH, 'Whether or not our disease would develop into PCa?'. Facing these questions, we often told them that prostatitis, BPH and PCa had nothing to do with each other, due to the absence of definite evidence. However, accumulating epidemiological studies have revealed the significant associations among prostatitis, BPH and PCa risk. Nevertheless, controversies still exist, and no consensus has been achieved on this topic till now. Hence, we comprehensively searched online databases and conducted this meta-analysis to clarify their correlations.

## Materials and methods

### Literature search strategy

We comprehensively retrieved relevant studies about the relations among prostatitis, BPH and PCa from online databases PubMed, PMC, EMBASE and Web of Science, published before April 1st, 2019, 2018. Following keywords combined with the Medical Subject Headings (MeSH) items were utilized: “Prostatitis” or “Prostatitides” or “Chronic Pelvic Pain Syndrome” or “Chronic Bacterial Prostatitis” or “Acute Bacterial Prostatitis” or “Asymptomatic Inflammatory Prostatitis”, “Benign Prostatic Hyperplasia” or “BPH” or “Prostatic Hyperplasia” or “Prostatic Hypertrophy”, “Prostate Cancer” or “Prostate Neoplasm” or “prostate tumor” or “PCa”. Furthermore, potentially eligible studies were meticulously identified by checking the reference lists from relevant review studies.

### Inclusion and exclusion criteria

Included studies must meet the following criteria: (1) Used a case-control or cohort study design; (2) Evaluated the epidemiological association among prostatitis, BPH and PCa. (3) Presented concrete numbers of exposures and non-exposures in both case and control groups to calculate the pooled odds ratios (ORs) and 95% confidence intervals (CIs); (4) Enrolled patients with PCa were confirmed by histopathological examination. In addition, exclusive criteria were as follows: (1) Not case-control or cohort studies; (2): Cross-sectional studies; (3) Duplicated studies or invalid data; (4) Studies not related to prostatitis, BPH and PCa.

### Data Extraction and Quality Assessment

All available data from the included studies were extracted independently by two reviewers (L.Z and Y.W) and summarized together. A third reviewer (ZQ.Q) would join in the discussion if any divergence arose and then reached a consensus. Finally, the extracted data were recorded in a standardized format including following items: first author's name, publication year, age of subjects, country, ethnicity, source of controls, study design, data source, the number of cases and controls, and the number of exposures. In addition, the quality of included studies was assessed with the Newcastle-Ottawa Scale (NOS). If the final score > 6, it was regarded as high-quality and then included in the subsequent meta-analyses.

### Statistical Analysis

Pooled ORs with 95% CIs were respectively calculated to evaluate the associations between prostatitis and PCa, BPH and PCa, prostatitis and BPH. Heterogeneity was tested by Cochrane Q test and Higgins *I^2^* statistic. If the heterogeneity was acceptable (*I^2^*<50% or P>0.10), the fixed effect model (a Mantel-Haenszel method) was adopted. Contrarily, the random effect model (a DerSimonian-Laird method) would be applied if heterogeneity was significant (*I^2^*>50% or* P*<0.10). Subgroup analyses were performed by ethnicity, study design, source of control (SOC), and sample size. Furthermore, the stability and reliability of the results was examined by sensitive analyses. The publication bias was assessed by Begg's funnel plots and Egger's linear regression test. A significant bias would be considered if the *P*<0.05. All data were processed by Stata software 12.0 (StataCorp LP, College Station, TX).

## Results

### Studies characteristics

**Figure 1:**
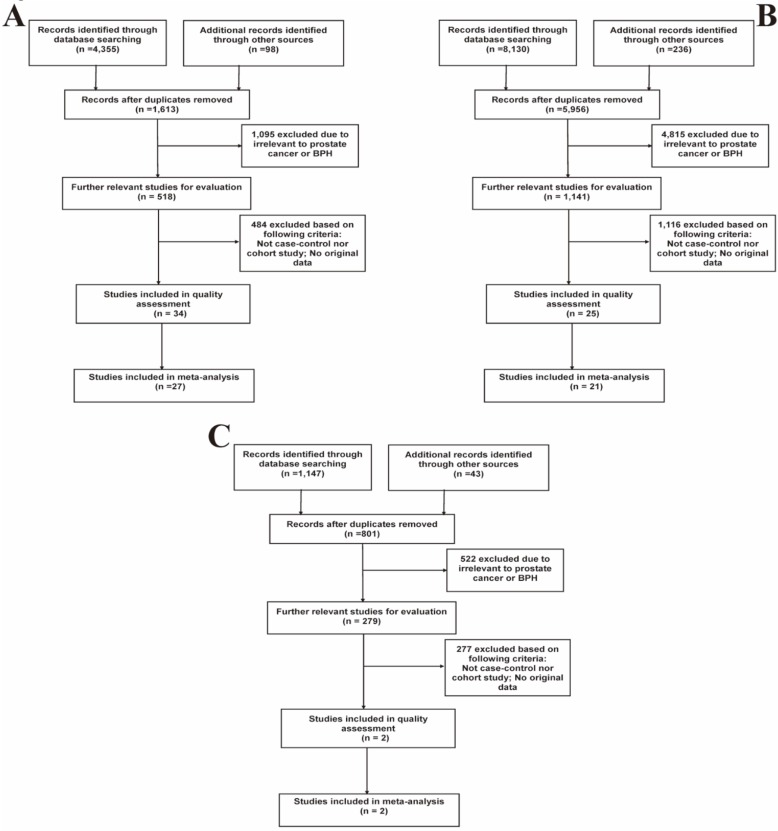
Flow diagrams of the literature selection process. **(A)** Prostatitis and PCa; **(B)** BPH and PCa; **(C)** Prostatitis and BPH.

Based on the above-mentioned inclusion and exclusion criteria, a total of 42 studies (S1-S42, **Table S1**) were included in the quality assessment. Results of the quality assessment were shown in **Table S2**. Ultimately, 35 high-quality studies with NOS scores > 6 were selected for further meta-analyses, while 7 studies (S36-S42) were eliminated. Among them, 27 studies described the relation between prostatitis and PCa, 21 eligible studies focused on the association between BPH and PCa, and 2 studies depicted the relation of prostatitis and BPH. **Figure 1** presented the specific details of searching literature and screening steps. The main characteristics of all included studies were separately listed in **Table 1**, **2** and** 3**.

### Association between prostatitis and PCa

**Figure 2:**
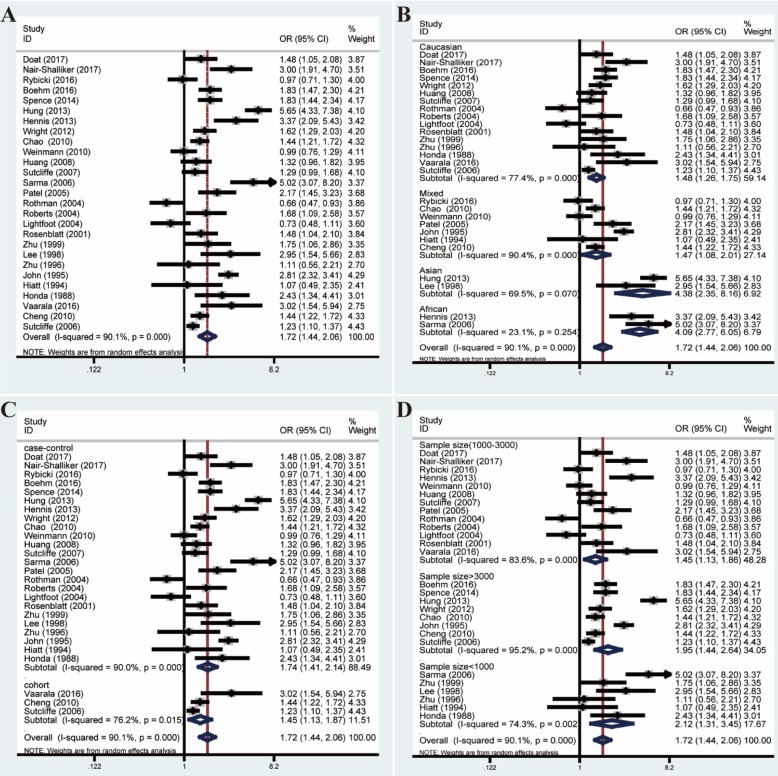
Forest plots of association between prostatitis and PCa by analyzing all enrolled studies. **(A)** Overall analysis; **(B)** The subgroup analyses of ethnicity; **(C)** study design; **(D)** sample size.

Pooled ORs and 95% CIs were calculated to assess the association between prostatitis and PCa. As shown in **Figure 2**, the overall analysis revealed a significant association between prostatitis and PCa (OR=1.72, 95% CI=1.44-2.06). However, a huge heterogeneity was detected in the results (*I²*=90.1%, *P*<0.001). Subsequent subgroup analyses failed to decrease the heterogeneity. Moreover, meta-regression analysis revealed the country and ethnicity might explain a certain proportion of the heterogeneity (Table S3). However, the heterogeneity remained remarkable when stratified by ethnicity.

**Figure 3:**
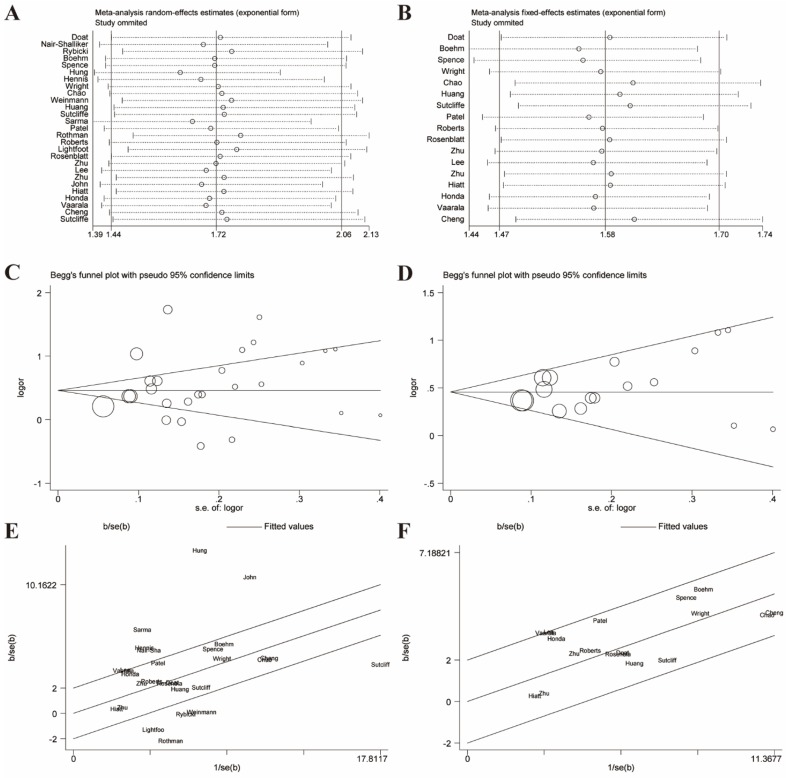
Galbraith plot associated with funnel plot and sensitive analysis of the association between prostatitis and PCa. **(A)** Sensitive analysis before adjustment for heterogeneity; **(B)** Funnel plot before adjustment for heterogeneity; **(C)** Galbraith plot before adjustment for heterogeneity; **(D)** Sensitive analysis after adjustment for heterogeneity; **(E)** Funnel plot after adjustment for heterogeneity; **(F)** Galbraith plot after adjustment for heterogeneity.

**Figure 4:**
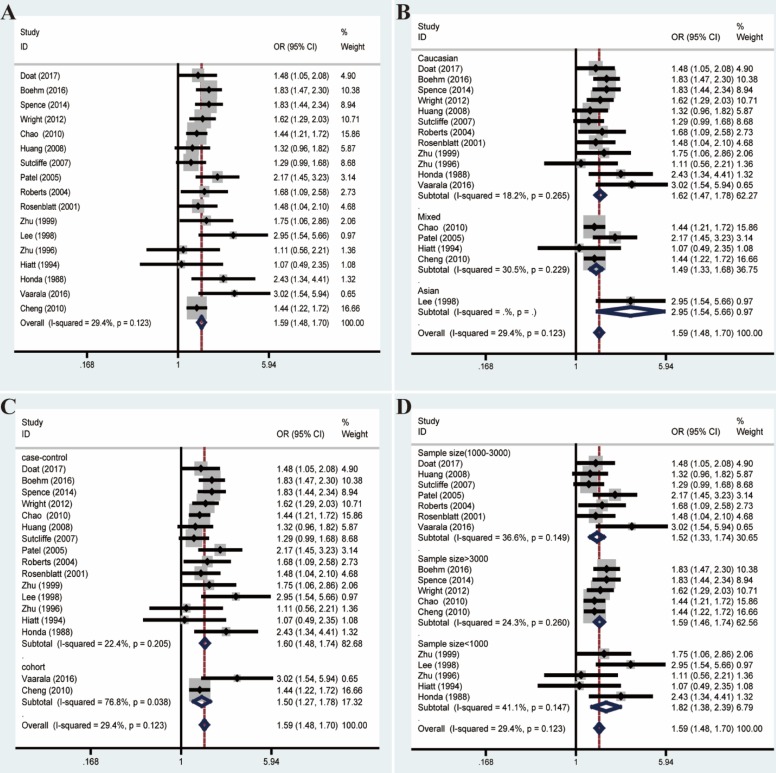
Forest plots of association between prostatitis and PCa after adjustment for heterogeneity based on Galbraith plot associated with funnel plot and sensitive analysis. **(A)** Overall analysis; **(B)** The subgroup analyses of ethnicity; **(C)** study design; **(D)** sample size.

Given these, we performed Galbraith radial plot to spot the outliers as the potential sources of heterogeneity (**Figure 3**). After eliminating partly of the studies according to the Galbraith plot, 17 residual studies were re-analyzed. New results remained significant (OR=1.59, 95% CI=1.48-1.70), and the overall heterogeneity was successfully decreased (*I²*=29.4%, *P*=0.123). What's more, subgroup analyses including ethnicity, study design and sample size showed significant results, which further validated this association (**Figure 4**).

### Association between BPH and PCa

**Figure 5:**
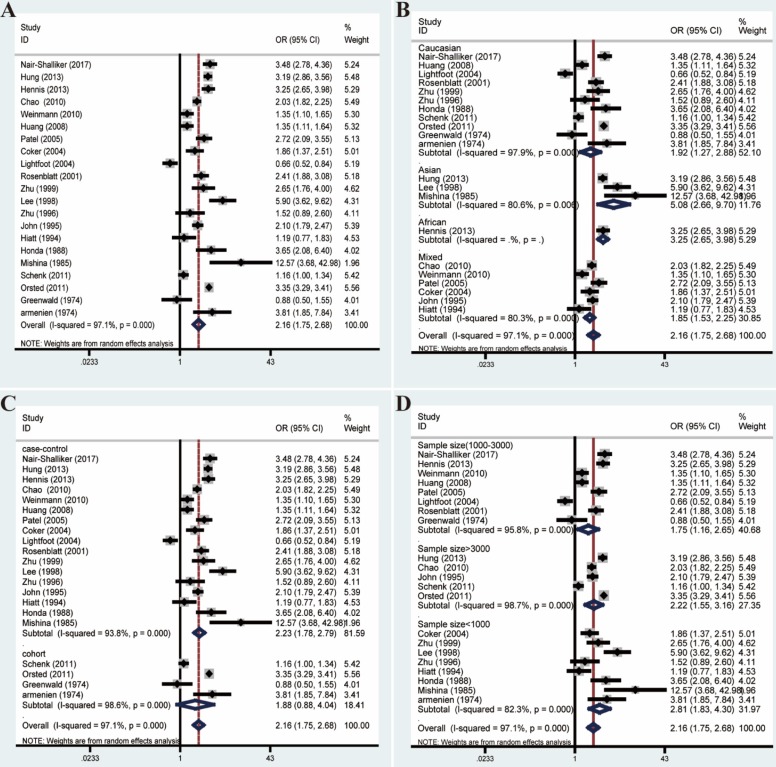
Forest plots of association between BPH and PCa by analyzing all enrolled studies. **(A)** Overall analysis; **(B)** The subgroup analyses of ethnicity; **(C)** study design; **(D)** sample size.

The overall analysis revealed a conspicuous association between BPH and PCa (OR=2.16, 95% CI=1.75-2.68). However, the great heterogeneity (*I²*=97.1%, *P*<0.001) also appeared and could not be reduced by subgroup analyses (**Figure 5**). What's more, meta-regression analyses suggested that no relevant covariates which could be summarized based on the between-study generality and individuality, could explain even part of the heterogeneity (**Table S3**).

**Figure 6:**
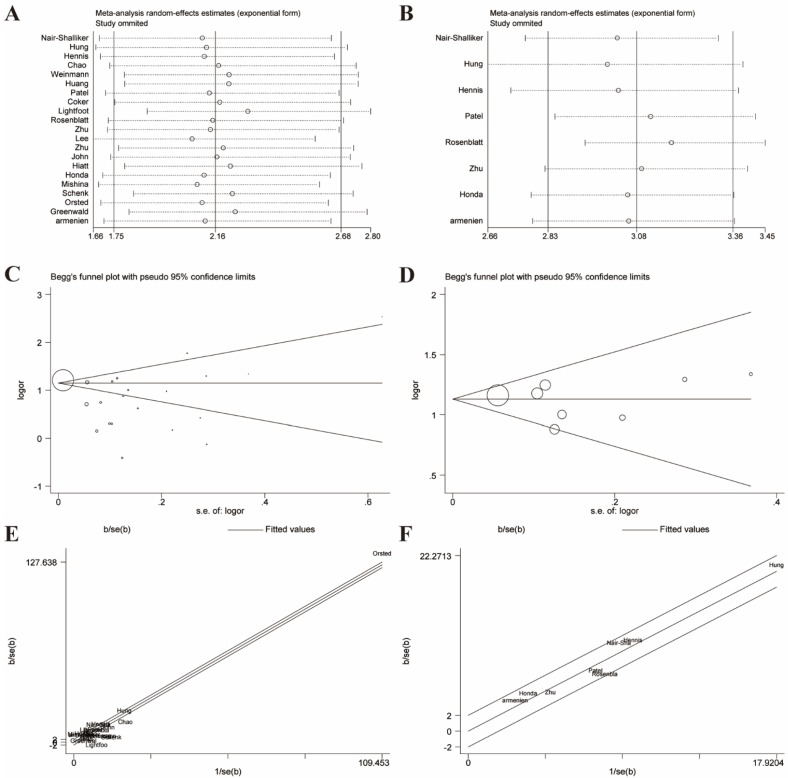
Galbraith plot associated with funnel plot and sensitive analysis of the association between BPH and PCa. **(A)** Sensitive analysis before adjustment for heterogeneity; **(B)** Funnel plot before adjustment for heterogeneity; **(C)** Galbraith plot before adjustment for heterogeneity; **(D)** Sensitive analysis after adjustment for heterogeneity; **(E)** Funnel plot after adjustment for heterogeneity; **(F)** Galbraith plot after adjustment for heterogeneity;

**Figure 7:**
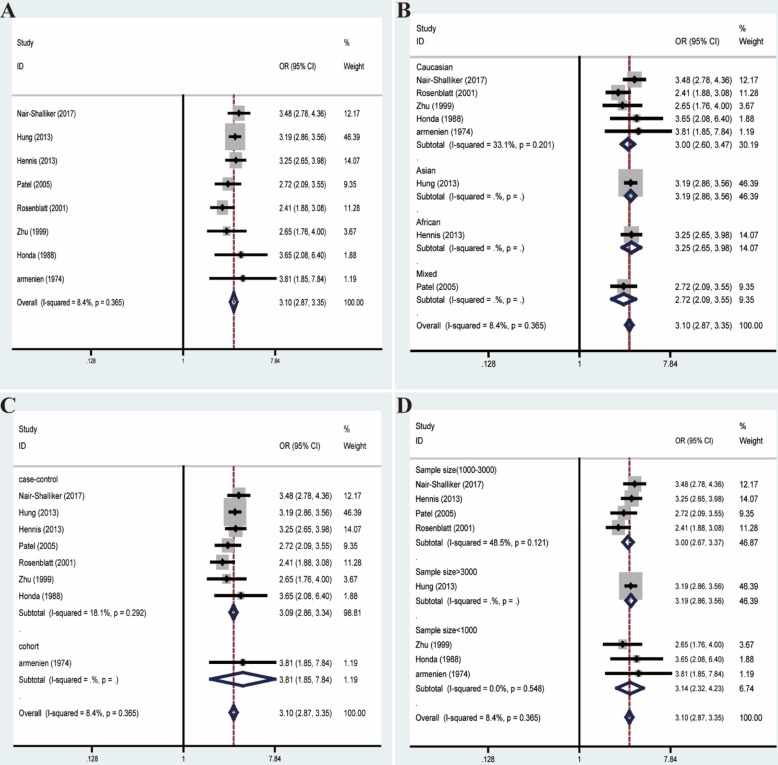
Forest plots of association between BPH and PCa after adjustment for heterogeneity based on Galbraith plot associated with funnel plot and sensitive analysis. **(A)** Overall analysis; **(B)** The subgroup analyses of ethnicity; **(C)** study design; **(D)** sample size.

Likewise, we excluded studies with distinct heterogeneity based on Galbraith plot in combination with results of Begg's funnel plot and sensitive analysis (**Figure 6**). Ultimately, post-elimination results of overall analysis (OR=3.10, 95% CI=2.87-3.35, *I²*=8.4%, *P*=0.365) and subgroup analyses maintained positive, which illustrated the significant association between BPH and PCa (**Figure 7**).

### Association between prostatitis and BPH

**Figure 8:**
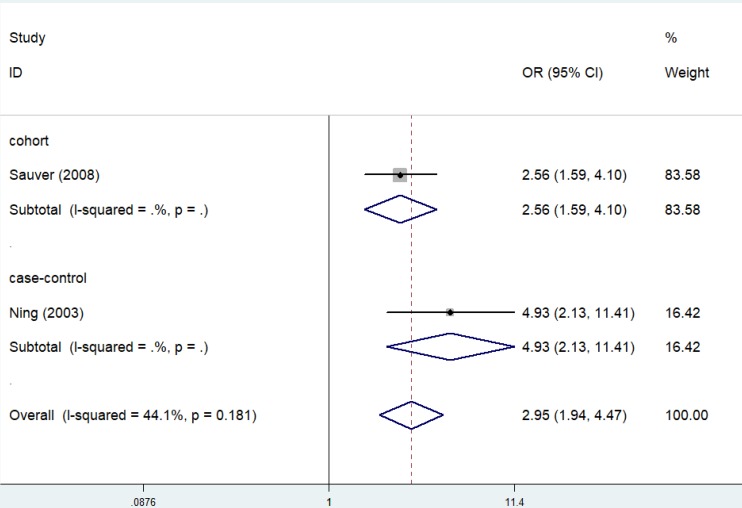
Forest plots of association between prostatitis and BPH.

Case-control studies and a cohort studies described the epidemiological relation of prostatitis with BPH. Significant results were obtained in the overall analysis (OR=2.95, 95% CI=1.94-4.47, *I²*=44.1%, *P*=0.181) (**Figure 8**). In addition, both the case-control study (OR=4.93, 95% CI=2.13-11.41) and the cohort study (OR=2.56, 95% CI=1.59-4.10) supported that prostatitis could enhance the risk of BPH.

### Sensitivity analysis

Sensitivity analyses were performed to evaluate the stability of results and reflect the impact of the individual study to overall results by deleting each study once a time. Our results indicated that no single study significantly influenced the pooled ORs and 95% CIs. Sensitivity analyses of the relation between prostatitis or BPH and PCa were respectively presented in** Figure 3A, 3B, 6A, 6B.**

### Publication bias

The Begg's funnel plot and Egger's test were performed to assess the publication bias. In the pooled analysis of prostatitis and PCa, Egger's *P* value was 0.17 and Begg's *P* value was 0.09 after dealing with the heterogeneity (**Figure 3E**). In the pooled analysis of BPH and PCa, Egger's *P* value was 0.763 and Begg's *P* value was 0.902 after elimination. (**Figure 6E**). Therefore, the original studies included in the present meta-analysis have no obvious publication bias.

## Discussion

Clinically, urologic physicians always asked by some anxious patients whether prostatitis or BPH will develop into PCa or increase the risk of PCa. However, there are no explicit answers on these questions to date. Herein, we carefully searched available literature to seek for explanations. Although no convincingly pathological evidence, a large number of epidemiological studies have revealed the close associations between prostatitis, BPH and PCa 27-29. However, no consensus has been reached. Hence, this meta-analysis was conducted to further clarify their triadic relationships by comprehensively summarizing the epidemiological studies.

As indicated in the results, prostatitis and BPH were both associated with escalating risks of PCa. Moreover, people with a history of prostatitis might be more vulnerable to BPH. All the results of subgroup analyses were positive. However, huge heterogeneity was existed among enrolled studies. Meta-regression results suggested only the country and ethnicity could o explain small parts of the heterogeneity. After careful analysis of all available data, the potential sources of the heterogeneity were displayed below. First, due to the extreme correlations of these three diseases with age, the different distribution of participants' age was regarded as a vital source of the heterogeneity. On the other hand, prostatitis could be divided into four categories: acute bacterial prostatitis, chronic bacterial prostatitis, Chronic Prostatitis/Chronic Pelvic Pain Syndrome (CP/CPPS) and asymptomatic inflammatory prostatitis, according to the National Institutes of Health (NIH) 30. Among included studies, no specific classification of prostatitis was identified, which might lead to inhomogeneity. On the other hand, the years of prostatitis or BPH history before getting PCa also affect the results to a great extent, while a good deal of studies had not paid attention to it. Rothman et al. found a highest relative risk for prostate cancer in men who had prostatitis diagnosed within 12 months of their prostate cancer reference data 31. Moreover, pathological types of PCa were also rarely mention in these studies. Furthermore, methodological heterogeneity and other various factors such as ways of getting data from participants, bias of participants' memories of medical history and differences of interviewers' emphasis all inevitably contributed to the source of the heterogeneity.

Galbraith plot as one way of displaying several estimates of the same quantity having different standard errors, was also used to spot the outlier as the possibly major source of between-study heterogeneity 32, 33. Thus, Galbraith plot associated with funnel plot and sensitive analysis was applied to filter those papers with higher heterogeneity and tried to pool analysis with those of approximately homogeneous papers. After carefully screening by the Galbraith plot and considering results from sensitive analyses as well as Begg's funnel plots together, 17 studies (*I^2^*=29.4%, *P*=0.123) with low heterogeneity were ultimately re-analyzed to display the relationship of prostatitis and PCa, and 8 studies were to re-analyzed the association between BPH and PCa (*I^2^*=8.4%, *P*=0.365). Finally, the results of overall and subgroup analyses remained significant. As for studies about prostatitis and BPH, although large heterogeneity existed as well, pooled OR with 95% CI of each independent study was all above 1, indicating that prostatitis could enhance the risk of BPH anyway.

Notably, the outcomes of us remained consistent before and after adjustment for heterogeneity and meanwhile positive results were obtained in both the overall and subgroup analyses, indicating the stability and reliability of our results. With the increasing recognition of the early-diagnosis and early-treatment of PCa, it had caught more and more attention in the recent years 34. Although several factors had been verified associated with the carcinogenesis of PCa involving aging, family history, race and altered androgen metabolism, its definite pathogenesis remained unclear 3-5. While the views “prostatitis could lead to higher PCa risk” or “BPH could increase the PCa susceptibility” had not been acknowledged, a massive number of epidemiological studies had been carried to explore whether prostatitis and BPH were risk factors of PCa. The latest cohort study with 2500 participants suggested that higher proportion in men with prostatitis were diagnosed with PCa after 15 years later 35. Besides, a cohort study conducted in Denmark with more than a hundred thousand participants from 1980 through 2006 also demonstrated that the incidence rate was higher in the BPH cohort 28. According to above results, the associations between prostatitis or BPH and PCa were almost conclusive.

Some potential hypotheses had been put forward in the decades, regarding the correlations between prostatitis, BPH and PCa. Inflammation was seen as highly correlated with several types of cancers including colon, stomach, liver and bladder 36-39. Chronic Inflammatory stimulating could induce various chemokines and cytokines generation that provided a favorable microenvironment for tumor growth and tumor progression by facilitating angiogenesis and increasing the production of reactive oxygen species (ROS), which can lead to oxidative DNA damage and reduced DNA repair 40. Chronic inflammatory lesions could be commonly detected in PCa patients when carrying out prostate biopsy 41. Jiang et al. conducted a meta-analysis of 20 case-control studies and found a significant positive relationship between prostatitis and PCa 12. Herein, we included larger numbers of studies and demonstrated a likewise significant relationship of prostatitis with PCa. However, which type of prostatitis was more inclined to associate with a higher risk of PCa remained to be determined.

As for BPH and PCa, strong arguments that BPH and PCa were unrelated, have been insisted in most urological surgeons on account of differences in the histologic and anatomic location of these 2 conditions that BPH mostly occurs in transitional zone of prostate while PCa often happen in peripheral zone 42. Besides, BPH was primarily characterized by hyperplasia of stromal, whereas PCa predominantly involves in the epithelium 42. Nonetheless, parallel features existed in the two diseases like hormone-dependent growth, response to androgen-deprivation treatments and relation with old age 43. Whether or not BPH could affect tumorigenesis via reacting to epithelium-stromal, it remained undetermined 44. On the other hand, plenty of studies investigated the epidemiological relationship between BPH and PCa, and found a positive effect of BPH on PCa risk 28, 45. Accordingly, we pooled all the results of available case-control or cohort studies to further shed light on their relationship and the results showed significant relation. However, those patients with a history of BPH were more likely to consult urologic physicians and perform a regular examination. As a result, increased the detection rate of PCa was found and this might lead to detection bias compared with healthy controls.

Our results suggested that prostatitis and BPH could increase the risk of PCa. Then, what's the connection between prostatitis and BPH? Nunzio et al. found common inflammatory infiltrates in BPH lesions and those cytokines and growth factors released by inflammatory cells could stimulate the stroma and epithelial cells to hyperproliferation 46. Taoka et al. reported that asymptomatic histological inflammation could induce repeated damage, repair, and regeneration of the prostate tissue, causing prostatic hyperplasia and leading to morphological changes of stromal tissue, which could increase urination resistance and result in symptomatic BPH 47. Nevertheless, whether prostatitis could lead to BPH has not been widely approved by urological specialists. Hence, we searched for eligible epidemiological studies to illuminate the potential relationship. A large number of cross-sectional studies have suggested that prostatitis and BPH are closely related with each other 48-50. A total of 1 case-control study and 1 cohort study were included in meta-analysis meeting the inclusion criteria. It was found that the BPH cases were more likely exposed to a prostatitis history than non-BPH controls 51. Furthermore, Sauver and his colleague conducted a 14-years follow-up cohort study and demonstrated the longitudinal association between prostatitis and development of BPH 26.

There were mainly four advantages in this article. On the one hand, this study was the first time to explore the associations between prostatitis, BPH and PCa at the same time from an epidemiological perspective and significant results were acquired. On the other hand, our study was performed with the extremely strict inclusion criteria which eliminating the cross-section studies in some surveys. Moreover, we performed strict quality evaluation by excluding low-quality studies (NOS<7) while the previous reviews did not focus on the quality evaluation. What's more, we preformed in-depth statistical analysis and careful comparison of multiple heterogeneous studies and tried our best to discussed the source of heterogeneity. However, the previous studies of meta-analysis describing similar topic did not detailly explain the source of the heterogeneity and deal with it. Herein, we firstly applied the approach of Galbraith plot to exclude the outlier-studies and compared the results pre and post-elimination. Thus, the integrated results are bound to elevated the reliability of our conclusions.

To a certain degree, several limitations of this paper should be considered: Firstly, the results were based on unadjusted estimates without modifying the influences of some other covariates like age and race; Secondly, the heterogeneity among enrolled studies was so huge that we have to eliminate some studies with higher heterogeneity and we could not present more detailed subgroup after analyzing the possible source of heterogeneity; Thirdly, prostatitis, BPH and PCa were all multifactorial diseases that other factors like age, environment, lifestyle and inheritance should also be taken into account as a whole. Fourthly, most included studies were case-control study which were less reliable than cohort studies. Thus, more high-quality cohort studies were required to shed light on the association between prostatitis, BPH and PCa. Last but not least, this paper illustrated their triadic relationships only from the epidemiological perspective and could not clarify whether or not progressive associations existed among prostatitis, BPH and PCa, as hepatitis, liver cirrhosis and hepatocellular carcinoma did.

## Supplementary Material

Supplementary figures and tables.

## Figures and Tables

**Table 1:** Main characteristics of eligible studies explore the association between prostatitis and prostate cancer.

Author	Year	Age	Country	Ethnicity	SOC	Design	Data source	No. cancer cases	No. controls	No. prostatitis in cases	No. prostatitis in controls
*Doat^S1^	2017	40-75	France	Caucasian	PB	case-control	interview	819	879	84	63
Nair-Shalliker^S2^	2017	19-94	Australia	Caucasian	PB	case-control	interview	1181	875	97	25
Rybicki^S3^	2016	NA	US	Mixed	PB	case-control	Medical record	574	574	102	105
*Boehm^S4^	2016	<76	Canada	Caucasian	PB	case-control	interview	1884	1965	223	134
*Spence^S5^	2014	40-79	Canada	Caucasian	PB	case-control	interview	1555	1586	195	115
Hung^S6^	2013	>50	China	Asian	PB	case-control	Medical record	1184	4376	137	99
Hennis^S7^	2013	NA	Barbadian	African	PB	case-control	interview	963	941	75	23
*Wright^S8^	2012	34-74	US	Caucasian	PB	case-control	interview	1754	1645	217	132
*Chao^S9^	2010	45-69	US	Mixed	PB	case-control	interview	1559	75384	139	4788
Weinmann^S10^	2010	45-84	US	Mixed	PB	case-control	Medical record	768	929	119	145
*Huang^S11^	2008	61-69	US	Caucasian	PB	case-control	interview	868	1283	78	89
*Sutcliffe^S12^	2007	40-75	US	Caucasian	PB	case-control	interview	691	691	152	124
Sarma^S13^	2006	40-79	US	African	PB	case-control	interview	129	706	34	47
*Patel^S14^	2005	50-74	US	Mixed	PB	case-control	interview	700	604	86	38
Rothman^S15^	2004	40-64	US	Caucasian	PB	case-control	interview	750	702	660	644
*Roberts^S16^	2004	63-77	US	Caucasian	PB	case-control	Medical record	409	803	41	50
Lightfoot^S17^	2004	45-84	Canada	Caucasian	PB	case-control	interview	760	1632	30	88
*Rosenblatt^S18^	2001	40-64	US	Caucasian	PB	case-control	interview	753	703	87	57
*Zhu^S19^	1999	40-69	US	Caucasian	PB	case-control	interview	159	277	37	41
*Lee^S20^	1998	45-89	China	Asian	PB	case-control	interview	133	265	32	16
*Zhu^S21^	1996	40-69	US	Caucasian	PB	case-control	interview	175	258	15	22
John^S22^	1995	<85	US	Mixed	PB	case-control	interview	1642	1636	418	177
*Hiatt^S23^	1994	NA	US	Mixed	PB	case-control	interview	177	177	14	13
*Honda^S24^	1988	<60	US	Caucasian	PB	case-control	interview	211	211	39	18
*Vaarala^S25^	2016	20-80	Finland	Caucasian	PB	cohort	interview	40	1732	13	238
*Cheng^S26^	2010	45-69	US	Mixed	PB	cohort	interview	1631	63613	147	4081
Sutcliffe^S27^	2006	40-75	US	Caucasian	PB	cohort	interview	2230	33356	421	5311

NA: Not available; SOC: Source of controls; PB: Population-based; HB: Hospital-based;^ S#^: Reference. *Adjustment for heterogeneity performed by excluding relevant studies as the outliers spotted by Galbraith plot and the possible major source of heterogeneity.

**Table 2:** Main characteristics of eligible studies explore the association between BPH and prostate cancer;

Author	Year	Age	Country	Ethnicity	SOC	Design	Data source	No. cancer cases	No. controls	No. BPH in cases	No. BPH in controls
*Nair-Shalliker^S2^	2017	19-94	Australia	Caucasian	PB	case-control	interview	1181	875	436	124
*Hung^S6^	2013	>50	China	Asian	PB	case-control	Medical record	1184	4763	1084	1071
*Hennis^S7^	2013	NA	Barbadian	African	PB	case-control	interview	963	941	428	186
Chao^S9^	2010	45-69	US	Mixed	PB	case-control	interview	1559	75384	514	14728
Weinmann^S10^	2010	45-84	US	Mixed	PB	case-control	Medical record	768	929	284	282
Huang^S11^	2008	61-69	US	Caucasian	PB	case-control	interview	868	1283	258	306
*Patel^S14^	2005	50-74	US	Mixed	PB	case-control	interview	700	604	246	101
Coker^S28^	2004	65-79	US	Mixed	PB	case-control	interview	407	393	159	102
Lightfoot^S17^	2004	45-84	Canada	Caucasian	PB	case-control	interview	710	1543	103	315
*Rosenblatt^S18^	2001	40-64	US	Caucasian	PB	case-control	interview	753	703	253	122
*Zhu^S19^	1999	40-69	US	Caucasian	PB	case-control	interview	156	281	77	75
Lee^S20^	1998	45-89	China	Asian	HB	case-control	interview	133	265	64	36
Zhu^S21^	1996	40-69	US	Caucasian	PB	case-control	interview	175	258	30	35
John^S22^	1995	<85	US	Mixed	PB	case-control	interview	1642	1636	539	309
Hiatt^S23^	1994	NA	US	Mixed	PB	case-control	interview	177	177	68	61
*Honda^S24^	1988	<60	US	Caucasian	PB	case-control	interview	211	211	56	19
Mishina^S29^	1985	45-89	Janpan	Asian	PB	case-control	interview	100	100	28	3
Schenk^S30^	2011	≥55	US	Caucasian	PB	cohort	interview	1225	2618	394	761
Ørsted^S31^	2011	20-100	Denmark	Caucasian	PB	cohort	interview	53171	794616	24486	161489
Greenwald^S32^	1974	<80	US	Caucasian	HB	cohort	Medical record	50	1590	24	814
*Armenien^S33^	1974	NA	US	Caucasian	HB	cohort	Medical record	45	566	35	271

NA: Not available; SOC: Source of controls; PB: Population-based; HB: Hospital-based; ^S#^: Reference. *Adjustment for heterogeneity performed by excluding relevant studies as the outliers spotted by Galbraith plot and the possible major source of heterogeneity.

**Table 3:** Main characteristics of eligible studies explore the association between prostatitis and BPH;

Author	Year	Age	Country	Ethnicity	SOC	Design	Data source	No. BPH cases	No. controls	No. prostatitis in cases	No. prostatitis in controls
Sauver^S34^	2008	40-79	US	Caucasian	cohort	PB	Medical record	1921	527	176	20
Ning^S35^	2003	60+	China	Asian	case-control	PB	interview	100	100	30	8

NA: Not available; SOC: Source of controls; PB: Population-based; HB: Hospital-based; ^S#^: Reference.

## References

[1] Siegel RL, Miller KD, Jemal A (2018). Cancer statistics, 2018. CA Cancer J Clin.

[2] Perdana NR, Mochtar CA, Umbas R, Hamid AR (2016). The Risk Factors of Prostate Cancer and Its Prevention: A Literature Review. Acta Med Indones.

[3] Bechis SK, Carroll PR, Cooperberg MR (2011). Impact of age at diagnosis on prostate cancer treatment and survival. J Clin Oncol.

[4] Kicinski M, Vangronsveld J, Nawrot TS (2011). An epidemiological reappraisal of the familial aggregation of prostate cancer: a meta-analysis. PLoS One.

[5] Rebbeck TR (2017). Prostate Cancer Genetics: Variation by Race, Ethnicity, and Geography. Semin Radiat Oncol.

[6] Ide H, Horie S (2011). Role of androgen in prostate carcinogenesis. Nihon Rinsho.

[7] Pukkala E, Martinsen JI, Lynge E, Gunnarsdottir HK, Sparen P, Tryggvadottir L (2009). Occupation and cancer - follow-up of 15 million people in five Nordic countries. Acta Oncol.

[8] De Nunzio C, Andriole GL, Thompson IJ, Freedland SJ (2015). Smoking and Prostate Cancer: A Systematic Review. Eur Urol Focus.

[9] Di Francesco S, Tenaglia RL (2014). Obesity, diabetes and aggressive prostate cancer hormone-naive at initial diagnosis. Cent European J Urol.

[10] Rizos C, Papassava M, Golias C, Charalabopoulos K (2010). Alcohol consumption and prostate cancer: a mini review. Exp Oncol.

[11] Brookman-May SD, Campi R, Henriquez J, Klatte T, Langenhuijsen JF, Brausi M (2018). Latest Evidence on the Impact of Smoking, Sports, and Sexual Activity as Modifiable Lifestyle Risk Factors for Prostate Cancer Incidence, Recurrence, and Progression: A Systematic Review of the Literature by the European Association of Urology Section of Oncological Urology (ESOU).

[12] Jiang J, Li J, Yunxia Z, Zhu H, Liu J, Pumill C (2013). The role of prostatitis in prostate cancer: meta-analysis. PLoS One.

[13] Dai X, Fang X, Ma Y, Xianyu J (2016). Benign Prostatic Hyperplasia and the Risk of Prostate Cancer and Bladder Cancer: A Meta-Analysis of Observational Studies. Medicine (Baltimore).

[14] Collins MM, Stafford RS, O'Leary MP, Barry MJ (1998). How common is prostatitis? A national survey of physician visits. J Urol.

[15] Mehik A, Hellstrom P, Lukkarinen O, Sarpola A, Jarvelin M (2000). Epidemiology of prostatitis in Finnish men: a population-based cross-sectional study. BJU Int.

[16] Tan JK, Png DJ, Liew LC, Li MK, Wong ML (2002). Prevalence of prostatitis-like symptoms in Singapore: a population-based study. Singapore Med J.

[17] Kryvenko ON, Jankowski M, Chitale DA, Tang D, Rundle A, Trudeau S (2012). Inflammation and preneoplastic lesions in benign prostate as risk factors for prostate cancer. Mod Pathol.

[18] Kim SH, Ha US, Yoon BI, Kim SW, Sohn DW, Kim HW (2014). Microbiological and clinical characteristics in acute bacterial prostatitis according to lower urinary tract manipulation procedure. J Infect Chemother.

[19] Ku JH, Paick JS, Kim SW (2005). Chronic prostatitis in Korea: a nationwide postal survey of practicing urologists in 2004. Asian J Androl.

[20] Khan FU, Ihsan AU, Nawaz W, Khan MZ, Yang M, Wang G (2017). A novel mouse model of chronic prostatitis/chronic pelvic pain syndrome induced by immunization of special peptide fragment with aluminum hydroxide adjuvant. Immunol Lett.

[21] McVary KT (2006). BPH: epidemiology and comorbidities. Am J Manag Care.

[22] Behre HM, Bohmeyer J, Nieschlag E (1994). Prostate volume in testosterone-treated and untreated hypogonadal men in comparison to age-matched normal controls. Clin Endocrinol (Oxf).

[23] Tavani A, Longoni E, Bosetti C, Maso LD, Polesel J, Montella M (2006). Intake of selected micronutrients and the risk of surgically treated benign prostatic hyperplasia: a case-control study from Italy. Eur Urol.

[24] Vignozzi L, Rastrelli G, Corona G, Gacci M, Forti G, Maggi M (2014). Benign prostatic hyperplasia: a new metabolic disease?. J Endocrinol Invest.

[25] Adorini L, Penna G, Fibbi B, Maggi M (2010). Vitamin D receptor agonists target static, dynamic, and inflammatory components of benign prostatic hyperplasia. Annals of the New York Academy of Sciences.

[26] St (2008). Sauver JL, Jacobson DJ, McGree ME, Girman CJ, Lieber MM, Jacobsen SJ. Longitudinal Association between Prostatitis and Development of Benign Prostatic Hyperplasia. Urology.

[27] Cheng I, Witte JS, Jacobsen SJ, Haque R, Quinn VP, Quesenberry CP (2010). Prostatitis, sexually transmitted diseases, and prostate cancer: the California Men's Health Study. PLoS One.

[28] Ørsted DD, Bojesen SE, Nielsen SF, Nordestgaard BG (2011). Association of Clinical Benign Prostate Hyperplasia with Prostate Cancer Incidence and Mortality Revisited: A Nationwide Cohort Study of 3 009 258 Men. European Urology.

[29] Nair-Shalliker V, Yap S, Nunez C, Egger S, Rodger J, Patel MI (2017). Adult body size, sexual history and adolescent sexual development, may predict risk of developing prostate cancer: Results from the New South Wales Lifestyle and Evaluation of Risk Study (CLEAR). Int J Cancer.

[30] Khan FU, Ihsan AU, Khan HU, Jana R, Wazir J, Khongorzul P (2017). Comprehensive overview of prostatitis. Biomed Pharmacother.

[31] Rothman I, Stanford JL, Kuniyuki A, Berger RE (2004). Self-report of prostatitis and its risk factors in a random sample of middle-aged men. Urology.

[32] Galbraith RF (1988). A note on graphical presentation of estimated odds ratios from several clinical trials. Stat Med.

[33] Galbraith RF (1988). Graphical Display of Estimates Having Differing Standard Errors. Technometrics.

[34] Shoag J, Mittal S, Halpern JA, Scherr D, Hu JC, Barbieri CE (2016). Lethal Prostate Cancer in the PLCO Cancer Screening Trial. Eur Urol.

[35] Vaarala MH, Mehik A, Ohtonen P, Hellstrom PA (2016). Prostate cancer incidence in men with self-reported prostatitis after 15 years of follow-up. Oncol Lett.

[36] Sinn DH, Lee J, Goo J, Kim K, Gwak GY, Paik YH (2015). Hepatocellular carcinoma risk in chronic hepatitis B virus-infected compensated cirrhosis patients with low viral load. Hepatology.

[37] Keller J, Chiou HY, Lin HC (2013). Increased risk of bladder cancer following diagnosis with bladder pain syndrome/interstitial cystitis. Neurourol Urodyn.

[38] Terzic J, Grivennikov S, Karin E, Karin M (2010). Inflammation and colon cancer. Gastroenterology.

[39] Matysiak-Budnik T, Megraud F (2006). Helicobacter pylori infection and gastric cancer. Eur J Cancer.

[40] Balkwill F, Mantovani A (2001). Inflammation and cancer: back to Virchow?. Lancet.

[41] Nickel JC, Roehrborn CG, O'Leary MP, Bostwick DG, Somerville MC, Rittmaster RS (2008). The relationship between prostate inflammation and lower urinary tract symptoms: examination of baseline data from the REDUCE trial. Eur Urol.

[42] De Marzo AM, Coffey DS, Nelson WG (1999). New concepts in tissue specificity for prostate cancer and benign prostatic hyperplasia. Urology.

[43] Bostwick DG, Cooner WH, Denis L, Jones GW, Scardino PT, Murphy GP (1992). The association of benign prostatic hyperplasia and cancer of the prostate. Cancer.

[44] Lee C (1996). Role of androgen in prostate growth and regression: stromal-epithelial interaction. Prostate Suppl.

[45] Schenk JM, Kristal AR, Arnold KB, Tangen CM, Neuhouser ML, Lin DW (2011). Association of Symptomatic Benign Prostatic Hyperplasia and Prostate Cancer: Results from the Prostate Cancer Prevention Trial. American Journal of Epidemiology.

[46] De Nunzio C, Kramer G, Marberger M, Montironi R, Nelson W, Schroder F (2011). The controversial relationship between benign prostatic hyperplasia and prostate cancer: the role of inflammation. Eur Urol.

[47] Taoka R, Kakehi Y (2017). The influence of asymptomatic inflammatory prostatitis on the onset and progression of lower urinary tract symptoms in men with histologic benign prostatic hyperplasia. Asian J Urol.

[48] Clemens JQ, Meenan RT, O Keeffe Rosetti MC, Kimes T, Calhoun EA (2007). Prevalence of and Risk Factors for Prostatitis: Population Based Assessment Using Physician Assigned Diagnoses. The Journal of Urology.

[49] Daniels NA, Ewing SK, Zmuda JM, Wilt TJ, Bauer DC (2005). Correlates and prevalence of prostatitis in a large community-based cohort of older men. Urology.

[50] Wallner LP, Clemens JQ, Sarma AV (2009). Prevalence of and risk factors for prostatitis in African American men: The Flint Men's Health Study. The Prostate.

[51] Ning X, Shi JP, Wu ZY, Zheng LG, Wang HL (2003). A case-control study on the risk factors of benign prostatic hyperplasia in the suburb of Shenyang. Zhonghua Liu Xing Bing Xue Za Zhi.

