# Supplementary material for: Correlation between Prostatitis, Benign Prostatic Hyperplasia and Prostate Cancer: A systematic review and Meta-analysis
Source: J Cancer. 2020 Jan 1;11(1):177–89. doi: 10.7150/jca.37235 (PMC6930406; doi:10.7150/jca.37235)
Supplement: Supplementary file 1 — Supplementary figures and tables. [file jcav11p0177s1.pdf]

Table S1: References of enrolled studies in this article.

S1: Doat, S., Cenee, S., Tretarre, B., Rebillard, X., Lamy, P.J., Bringer, J.P., et al. (2017). Nonsteroidal anti-inflammatory drugs (NSAIDs) and prostate cancer risk: results from the EPICAP study. *Cancer Med* 6(10)**,** 2461-2470. doi: 10.1002/cam4.1186.

S2: Nair-Shalliker, V., Yap, S., Nunez, C., Egger, S., Rodger, J., Patel, M.I., et al. (2017). Adult body size, sexual history and adolescent sexual development, may predict risk of developing prostate cancer: Results from the New South Wales Lifestyle and Evaluation of Risk Study (CLEAR). *Int J Cancer* 140(3)**,** 565-574. doi: 10.1002/ijc.30471.

S3: Rybicki, B.A., Kryvenko, O.N., Wang, Y., Jankowski, M., Trudeau, S., Chitale, D.A., et al. (2016). Racial differences in the relationship between clinical prostatitis, presence of inflammation in benign prostate and subsequent risk of prostate cancer. *Prostate Cancer Prostatic Dis* 19(2)**,** 145-150. doi: 10.1038/pcan.2015.54.

S4: Boehm, K., Valdivieso, R., Meskawi, M., Larcher, A., Schiffmann, J., Sun, M., et al. (2016). Prostatitis, other genitourinary infections and prostate cancer: results from a population-based case-control study. *World J Urol* 34(3)**,** 425-430. doi: 10.1007/s00345-015-1625-1.

S5: Spence, A.R., Rousseau, M.C., Karakiewicz, P.I., and Parent, M.E. (2014). Circumcision and prostate cancer: a population-based case-control study in Montreal, Canada. *BJU Int* 114(6b)**,** E90-E98. doi: 10.1111/bju.12741.

S6: Hung, S.-C., Lai, S.-W., Tsai, P.-Y., Chen, P.-C., Wu, H.-C., Lin, W.-H., et al. (2013). Synergistic interaction of benign prostatic hyperplasia and prostatitis on prostate cancer risk. *British Journal of Cancer* 108(9)**,** 1778-1783. doi: 10.1038/bjc.2013.184.

S7: Hennis, A.J., Wu, S.Y., Nemesure, B., and Leske, M.C. (2013). Urologic characteristics and sexual behaviors associated with prostate cancer in an african-Caribbean population in barbados, west indies. *Prostate Cancer* 2013**,** 682750. doi: 10.1155/2013/682750.

S8: Wright, J.L., Lin, D.W., and Stanford, J.L. (2012). Circumcision and the risk of prostate cancer. *Cancer* 118(18)**,** 4437-4443. doi: 10.1002/cncr.26653.

S9: Chao, C., Haque, R., Van Den Eeden, S.K., Caan, B.J., Poon, K.Y., and Quinn, V.P. (2010). Red wine consumption and risk of prostate cancer: the California men's health study. *Int J Cancer* 126(1)**,** 171-179. doi: 10.1002/ijc.24637.

S10: Weinmann, S., Shapiro, J.A., Rybicki, B.A., Enger, S.M., Van Den Eeden, S.K., Richert-Boe, K.E., et al. (2010). Medical history, body size, and cigarette smoking in relation to fatal prostate cancer. *Cancer Causes Control* 21(1)**,** 117-125. doi: 10.1007/s10552-009-9441-9.

S11: Huang, W.Y., Hayes, R., Pfeiffer, R., Viscidi, R.P., Lee, F.K., Wang, Y.F., et al. (2008). Sexually transmissible infections and prostate cancer risk. *Cancer Epidemiol Biomarkers Prev* 17(9)**,** 2374-2381. doi: 10.1158/1055-9965.EPI-08-0173.

S12: Sutcliffe, S., Giovannucci, E., Gaydos, C.A., Viscidi, R.P., Jenkins, F.J., Zenilman, J.M., et al. (2007). Plasma antibodies against Chlamydia trachomatis, human papillomavirus, and human herpesvirus type 8 in relation to prostate cancer: a prospective study. *Cancer Epidemiol Biomarkers Prev* 16(8)**,** 1573-1580. doi: 10.1158/1055-9965.EPI-07-0134.

S13: Sarma, A.V., McLaughlin, J.C., Wallner, L.P., Dunn, R.L., Cooney, K.A., Schottenfeld, D., et al. (2006). Sexual behavior, sexually transmitted diseases and prostatitis: the risk of prostate cancer in black men. *J Urol* 176(3)**,** 1108-1113. doi: 10.1016/j.juro.2006.04.075.

S14: Patel, D.A., Bock, C.H., Schwartz, K., Wenzlaff, A.S., Demers, R.Y., and Severson, R.K. (2005). Sexually transmitted diseases and other urogenital conditions as risk factors for prostate

cancer: a case–control study in Wayne County, Michigan. *Cancer Causes & Control* 16(3), 263-273. doi: 10.1007/s10552-004-3486-6.
S15: Rothman, I., Stanford, J.L., Kuniyuki, A., and Berger, R.E. (2004). Self-report of prostatitis and its risk factors in a random sample of middle-aged men. *Urology* 64(5), 876-879. doi: 10.1016/j.urology.2004.06.071.
S16: Roberts, R.O., Bergstralh, E.J., Bass, S.E., Lieber, M.M., and Jacobsen, S.J. (2004). Prostatitis as a risk factor for prostate cancer. *Epidemiology* 15(1), 93-99. doi: 10.1097/01.ede.0000101022.38330.7c.
S17: Lightfoot, N., Conlon, M., Kreiger, N., Sass-Kortsak, A., Purdham, J., and Darlington, G. (2004). Medical history, sexual, and maturational factors and prostate cancer risk. *Ann Epidemiol* 14(9), 655-662. doi: 10.1016/j.annepidem.2003.11.006.
S18: Rosenblatt, K.A., Wicklund, K.G., and Stanford, J.L. (2001). Sexual factors and the risk of prostate cancer. *Am J Epidemiol* 153(12), 1152-1158.
S19: Zhu, K., McKnight, B., Stergachis, A., Daling, J.R., and Levine, R.S. (1999). Comparison of self-report data and medical records data: results from a case-control study on prostate cancer. *Int J Epidemiol* 28(3), 409-417.
S20: Lee, M.M., Wang, R.T., Hsing, A.W., Gu, F.L., Wang, T., and Spitz, M. (1998). Case-control study of diet and prostate cancer in China. *Cancer Causes Control* 9(6), 545-552.
S21: Zhu, K., Stanford, J.L., Daling, J.R., McKnight, B., Stergachis, A., Brawer, M.K., et al. (1996). Vasectomy and prostate cancer: a case-control study in a health maintenance organization. *Am J Epidemiol* 144(8), 717-722.
S22: John, E.M., Whittemore, A.S., Wu, A.H., Kolonel, L.N., Hislop, T.G., Howe, G.R., et al. (1995). Vasectomy and prostate cancer: results from a multiethnic case-control study. *J Natl Cancer Inst* 87(9), 662-669.
S23: Hiatt, R.A., Armstrong, M.A., Klatsky, A.L., and Sidney, S. (1994). Alcohol consumption, smoking, and other risk factors and prostate cancer in a large health plan cohort in California (United States). *Cancer Causes Control* 5(1), 66-72.
S24: Honda, G.D., Bernstein, L., Ross, R.K., Greenland, S., Gerkins, V., and Henderson, B.E. (1988). Vasectomy, cigarette smoking, and age at first sexual intercourse as risk factors for prostate cancer in middle-aged men. *Br J Cancer* 57(3), 326-331.
S25: Vaarala, M.H., Mehik, A., Ohtonen, P., and Hellstrom, P.A. (2016). Prostate cancer incidence in men with self-reported prostatitis after 15 years of follow-up. *Oncol Lett* 12(2), 1149-1153. doi: 10.3892/ol.2016.4702.
S26: Cheng, I., Witte, J.S., Jacobsen, S.J., Haque, R., Quinn, V.P., Quesenberry, C.P., et al. (2010). Prostatitis, sexually transmitted diseases, and prostate cancer: the California Men's Health Study. *PLoS One* 5(1), e8736. doi: 10.1371/journal.pone.0008736.
S27: Sutcliffe, S., Giovannucci, E., De Marzo, A.M., Leitzmann, M.F., Willett, W.C., and Platz, E.A. (2006). Gonorrhea, syphilis, clinical prostatitis, and the risk of prostate cancer. *Cancer Epidemiol Biomarkers Prev* 15(11), 2160-2166. doi: 10.1158/1055-9965.EPI-05-0913.
S28: Coker, A.L., Sanderson, M., Zheng, W., and Fadden, M.K. (2004). Diabetes mellitus and prostate cancer risk among older men: population-based case-control study. *Br J Cancer* 90(11), 2171-2175. doi: 10.1038/sj.bjc.6601857.
S29: Mishina, T., Watanabe, H., Araki, H., and Nakao, M. (1986). Epidemiological Study of Prostatic Cancer by Matched-Pair Analysis. *The Journal of Urology* 135(3), 656. doi:

https://doi.org/10.1016/S0022-5347(17)45779-X.
S30: Schenk, J.M., Kristal, A.R., Arnold, K.B., Tangen, C.M., Neuhouser, M.L., Lin, D.W., et al. (2011). Association of Symptomatic Benign Prostatic Hyperplasia and Prostate Cancer: Results from the Prostate Cancer Prevention Trial. *American Journal of Epidemiology* 173(12)**,** 1419-1428. doi: 10.1093/aje/kwq493.
S31: Ørsted, D.D., Bojesen, S.E., Nielsen, S.F., and Nordestgaard, B.G. (2011). Association of Clinical Benign Prostate Hyperplasia with Prostate Cancer Incidence and Mortality Revisited: A Nationwide Cohort Study of 3 009 258 Men. *European Urology* 60(4)**,** 691-698. doi: 10.1016/j.eururo.2011.06.016.
S32: Greenwald, P., Kirmss, V., Polan, A.K., and Dick, V.S. (1974). Cancer of the prostate among men with benign prostatic hyperplasia. *J Natl Cancer Inst* 53(2)**,** 335-340.
S33: Armenian, H.K., Lilienfeld, A.M., Diamond, E.L., and Bross, I.D. (1974). Relation between benign prostatic hyperplasia and cancer of the prostate. A prospective and retrospective study. *Lancet* 2(7873)**,** 115-117.
S34: St. Sauver, J.L., Jacobson, D.J., McGree, M.E., Girman, C.J., Lieber, M.M., and Jacobsen, S.J. (2008). Longitudinal Association between Prostatitis and Development of Benign Prostatic Hyperplasia. *Urology* 71(3)**,** 475-479. doi: 10.1016/j.urology.2007.11.155.
S35: Ning, X., Shi, J.P., Wu, Z.Y., Zheng, L.G., and Wang, H.L. (2003). A case-control study on the risk factors of benign prostatic hyperplasia in the suburb of Shenyang. *Zhonghua Liu Xing Bing Xue Za Zhi* 24(4)**,** 276-280.
S36: Al Qadire, M., Alkhalaileh, M., and M, A.L. (2018). Lifestyle and Dietary Factors and Prostate Cancer Risk: A Multicentre Case-Control Study. *Clin Nurs Res***,** 1054773818757311. doi: 10.1177/1054773818757311.
S37: Hosseini, M., SeyedAlinaghi, S., Mahmoudi, M., and McFarland, W. (2010). A case-control study of risk factors for prostate cancer in Iran. *Acta Med Iran* 48(1)**,** 61-66.
S38: Daniels, N.A., Chen, Y.H., and Bent, S. (2009). Antibiotic and anti-inflammatory use and the risk of prostate cancer. *BMC Res Notes* 2**,** 57. doi: 10.1186/1756-0500-2-57.
S39: Genital and urinary tract diseases and prostate cancer risk. *Eur J Cancer Prev* 15(3)**,** 254-257. doi: 10.1097/01.cej.0000198897.40303.d4.
S40: Ritchie, J.M., Vial, S.L., Fuortes, L.J., Guo, H., Reedy, V.E., and Smith, E.M. (2003). Organochlorines and risk of prostate cancer. *J Occup Environ Med* 45(7)**,** 692-702. doi: 10.1097/01.jom.0000071510.96740.0b.
S41: Hsing, A.W., Wang, R.T., Gu, F.L., Lee, M., Wang, T., Leng, T.J., et al. (1994). Vasectomy and prostate cancer risk in China. *Cancer Epidemiol Biomarkers Prev* 3(4)**,** 285-288.
S42: Wei, Q., Tang, X., Yang, Y., Zhan, Y., and Yin, H. (1994). [Risk factors of prostate cancer--a matched case-control study]. *Hua Xi Yi Ke Da Xue Xue Bao* 25(1)**,** 87-90.

Table S2: Results of quality assessment;

1: Quality assessment of 34 individual studies on association between prostatitis and prostate cancer.

Case control study	SELECTION				Comparability	Exposure			
	Is the Case Definition Adequate?	Representativeness of the Cases	Selection of Controls	Definition of Controls	Comparability of cases and controls on the basis of the design or analysis	Ascertainment of exposure	Same method of ascertainment for cases and controls	Non-Response rate	Total
**Qadire2018**	*	*		*	*	*	*		**6**
**Doat2017**	*	*	*	*	*	*	*	*	**8**
**Nair-Shalliker2017**	*	*	*	*	**	*	*	*	**9**
**Rybicki2016**	*	*	*	*	*		*	*	**7**
**Boehm2016**	*	*	*	*	*		*	*	**7**
**Spence2014**	*	*	*	*	**	*	*		**8**
**Hung2013**	*	*	*	*	*	*	*	*	**8**
**Hennis2013**	*	*	*	*	*	*	*		**7**
**Wright2012**	*	*	*	*	*	*	*	*	**8**
**Hosseini2010**	*	*	*	*	*		*		**6**
**Chao2010**	*	*	*	*	*	*	*	*	**8**
**Weinmann2010**	*		*	*	*	*	*	*	**7**
**Daniels2009**	*		*		*	*	*		**5**
**Huang2008**	*	*	*	*	*		*	*	**7**
**Sutcliffe2007**	*	*	*	*	**		*		**7**
**Pelucchi2006**	*	*			*	*	*		**5**
**Sarma2006**	*	*	*	*	*	*	*		**7**
**Patel2005**	*	*	*	*	*		*	*	**7**
**Rothman2004**	*	*	*	*	*	*	*		**7**

**Roberts2004**	*	*	*	*	**	*	*		**8**
**Lightfoot2004**	*	*	*	*	*	*	*		**7**
**Ritchie2003**	*	*	*	*	*		*		**6**
**Rosenblatt2001**	*	*	*	*	*	*	*		**7**
**Zhu1999**	*	*	*	*	*		*	*	**7**
**Lee1998**	*	*	*	*	**		*		**7**
**Zhu1996**	*	*	*	*	*	*	*		**7**
**John1995**	*	*	*	*	*	*	*		**7**
**Hsing1994**	*	*		*	*	*	*		**6**
**Hiatt1994**	*		*	*	*	*	*	*	**7**
**Wei1994**	*			*	*	*	*		**5**
**Honda1988**	*	*	*	*	*	*	*		**7**
**Cohort study**	**SELECTION**				**Comparability**	**Outcome**			
	Representativeness of the exposed cohort	Selection of the non exposed cohort	Ascertainment of exposure	Demonstration that outcome of interest was not present at start of study	Comparability of cohorts on the basis of the design or analysis	Assessment of outcome	Was follow-up long enough for outcomes to occur	Adequacy of follow up of cohorts	Total
**Vaarala2016**	*	*	*	*	*	*	*	*	**8**
**Cheng2010**	*	*	*	*	*	*	*		**7**
**Sutcliffe2006**	*	*	*	*	**	*	*	*	**9**

2: Quality assessment of 25 individual studies on association between BPH and prostate cancer.

**Case control study**	**SELECTION**				**Comparability**	**Exposure**			
	Is the Case Definition Adequate?	Representativeness of the Cases	Selection of Controls	Definition of Controls	Comparability of cases and controls on the basis of the design or analysis	Ascertainment of exposure	Same method of ascertainment for cases and controls	Non-Response rate	Total
**Nair-Shalliker2017**	*	*	*	*	**	*	*	*	**9**
**Hung2013**	*	*	*	*	*	*	*	*	**8**
**Hennis2013**	*	*	*	*	*	*	*		**7**
**Chao2010**	*	*	*	*	*	*	*	*	**8**
**Weinmann2010**	*		*	*	*	*	*	*	**7**
**Daniels2009**	*		*		*	*	*		**5**
**Huang2008**	*	*	*	*	*		*	*	**7**
**Pelucchi2006**	*	*			*	*	*		**5**
**Patel2005**	*	*	*	*	*		*	*	**7**
**Coker2004**	*	*	*	*	**	*	*		**8**
**Lightfoot2004**	*	*	*	*	*	*	*		**7**
**Rosenblatt2001**	*	*	*	*	*	*	*		**7**
**Zhu1999**	*	*	*	*	*		*	*	**7**
**Lee1998**	*	*	*	*	**		*		**7**
**Zhu1996**	*	*	*	*	*	*	*		**7**
**John1995**	*	*	*	*	*	*	*		**7**
**Hsing1994**	*	*		*	*	*	*		**6**
**Hiatt1994**	*		*	*	*	*	*	*	**7**
**Wei1994**	*			*	*	*	*		**5**

**Honda1988**	*	*	*	*	*	*	*		**7**
**Mishina1985**	*	*	*	*	*	*	*		**7**
**Cohort study**	**SELECTION**				**Comparability**	**Outcome**			
	Representativeness of the exposed cohort	Selection of the non exposed cohort	Ascertainment of exposure	Demonstration that outcome of interest was not present at start of study	Comparability of cohorts on the basis of the design or analysis	Assessment of outcome	Was follow-up long enough for outcomes to occur	Adequacy of follow up of cohorts	Total
**Schenk2011**	*	*	*	*	**	*	*		**8**
**Ørsted2011**	*	*	*	*	**	*	*	*	**9**
**Greenwald1974**	*		*	*	**	*	*	*	**8**
**Armenien1974**	*		*	*	**	*	*	*	**8**

3: Quality assessment of 4 individual studies on association between prostatitis and BPH.

**Case control study**	**SELECTION**				**Comparability**	**Exposure**			
	Is the Case Definition Adequate?	Representativeness of the Cases	Selection of Controls	Definition of Controls	Comparability of cases and controls on the basis of the design or analysis	Ascertainment of exposure	Same method of ascertainment for cases and controls	Non-Response rate	Total
**Ning2003**	*	*	*		*	*	*	*	**7**
**Cohort study**	**SELECTION**				**Comparability**	**Outcome**			
	Representativeness of the exposed cohort	Selection of the non exposed cohort	Ascertainment of exposure	Demonstration that outcome of interest was not present at start of study	Comparability of cohorts on the basis of the design or analysis	Assessment of outcome	Was follow-up long enough for outcomes to occur	Adequacy of follow up of cohorts	Total
**Sauver2009**	*	*	*	*	*	*		*	**7**

Table S3: Results of meta-regression.

Meta Regression	Prostatitis and PCa	BPH and PCa
Covariate	P value	
Country	0.041	0.433
Ethnicity	0.003	0.904
Sources of exposure assessment	0.969	0.673
public year	0.79	0.618
Samplesize	0.124	0.44
Design	0.871	0.599
SOC	-	0.637
